# Obstructed Labor, Evolution, and Health Disparities

**DOI:** 10.3390/biology13121001

**Published:** 2024-12-01

**Authors:** Liliana Light, Suman Kaur Virdee, Colin Dickens, Rui Diogo

**Affiliations:** 1 College of Medicine, Howard University, Washington, DC 20001, USA; liliana.light@bison.howard.edu (L.L.); suman.virdee@bison.howard.edu (S.K.V.); colin.dickens@bison.howard.edu (C.D.); 2 Department of Anatomy, Howard University, Washington, DC 20059, USA

**Keywords:** obstructed labor, fetopelvic disproportion, obstetric dilemma, childbirth, obstetrical pelvis

## Abstract

The female pelvis is often evolutionarily described as a compromise to accommodate the birthing process and bipedalism. This compromise puts a mother and baby at risk of fetopelvic disproportion, the mismatch between the size of the fetus and that of the mother’s pelvis, impacting the ease with which the vaginal birthing process occurs. Obstructed labor, commonly caused by fetopelvic disproportion, is a leading cause of maternal mortality and morbidity and has serious medical sequelae for the fetus. In this review, this evolutionary aspect of fetopelvic disproportion is reconsidered within a broader sociocultural and environmental approach related to a change of paradigm from a more reductionist Neo-Darwinist to a more encompassing Extended Evolutionary Synthesis view. The review explores a more comprehensive understanding of several factors related to fetopelvic disproportion, including socioeconomic factors and ethnic disparities among individuals that might lead to a higher likelihood of obstructed labor and maternal and fetal morbidity and mortality.

## Simple Summary

The female pelvis has been described as being the result of a purely evolutionary tradeoff between giving birth and walking upright. However, it is now becoming clear that other important factors are also at play, including biocultural, socioeconomic, and genetic factors influencing female pelvis shape and size.

## 1. Introduction

The complexities of the human birthing process stem from the unique shape of the pelvis as a product of human evolution. The shape of the human female pelvis is described as a compromise, or evolutionary tradeoff, between a shape optimized for bipedal locomotion and a morphology optimized for birthing fetuses with relatively large heads due to an encephalization process that occurred during the last millions of years of our evolution. This tradeoff hypothesis, known as the “obstetric dilemma” (OD) hypothesis, was proposed by researchers such as Washburn: a large pelvis limits functional bipedalism, and a pelvis too narrow impedes an uncomplicated vaginal delivery [1]. That is, the assumption became that the female pelvis is a tradeoff between the two, settling for a size that allows bipedal walking and birthing without facing a serious disadvantage. If not for this compromise, birthing would not be a favorable process, leading to low fitness for the mother [2].

Medically, of the causes of maternal morbidity and mortality, pelvis size is often implicated in obstructed labor, which is a failure of labor to progress due to mechanical reasons. Fetopelvic disproportion is the primary cause of obstructed labor and is the ratio between the baby’s limiting factor in size, typically the head circumference or shoulder girdle width, and the mother’s pelvis [3]. The proposition of the OD hypothesis satisfied the reasoning behind fetopelvic disproportion. However, more recently, studies aiming to explain why fetopelvic disproportion occurs have identified other factors, including genetics, metabolic factors, and nutrition. These impact the mother’s and fetus’s physiology and anatomy, including rapid environmental changes, phenotypic plasticity, and biocultural factors [4,5,6]. In a sense, this is related to a very important change that has been occurring in the field of evolutionary biology in the past decades: more and more researchers—including some of the authors of this paper—are becoming part of a major change from a more reductionist and often genetic-centric Neo-Darwinian approach to a more encompassing view of life, often described as an Extended Evolutionary Synthesis, in which organismal behavior, niche construction, and epigenetics are seen as crucial aspects of evolution, together with genetics [7,8].

It is therefore important that evolutionary medicine, and, in particular, discussions on the causes of fetopelvic disproportion, start to take into consideration new ideas and factors, such as socioeconomic and ethnic disparities, as this might lead to a more comprehensive understanding of and, therefore, a potential decrease in the occurrence of obstructed labor and the need for surgical intervention during births. For instance, in recent years, there has been a significant increase in the percentage of babies that are born from cesarean sections (CSs) in numerous countries, including the U.S. [9]. Originally, this practice was mostly reserved for severe cases of obstructed labor, in which surgical intervention would replace vaginal birth to prevent the sequelae of the baby being halted in the birth canal, including asphyxiation, stillbirth, and neonatal jaundice [10]. However, the steady rise in cesarean section rates worldwide has been criticized, as this practice is primarily for problematic deliveries that could put both the mother and the baby at risk. The World Health Organization (WHO) recommends CS rates of 15% worldwide—that is, corresponding to the number that, on average, is truly needed to save the life of, or reduce risks to, the mother and/or baby: however, the yearly CS rate of all births has been as high as 32% in the U.S. and even higher in other countries worldwide, with Brazil’s rate being as high as 40%. In recent years, there has been a great disparity in the rate of CSs across ethnic groups, with African American women having significantly higher rates of CSs [9,11]. Disparities in cesarean section rates among Native American and Latino women are also influenced by socioeconomic factors, access to healthcare, and implicit biases within medical institutions. These factors contribute to a higher prevalence of cesarean deliveries in these communities, mirroring the trends observed in African American women [12]. Therefore, it is crucial and timely to have a new, more holistic perspective, aligning with recent developments and ideas in the fields of evolutionary biology and evolutionary medicine, when examining the very complex and intricate causes of fetopelvic disproportion. The main aim of this review is to provide, for the first time, a clear, succinct analysis of such factors, including genetics and nutrition, and explore how they contribute to ethnic disparities in obstructed labor to pave the way for more discussions on this important topic by academics, medical practitioners and researchers, and the broader public.

## 2. Evolution

**Figure 1:**
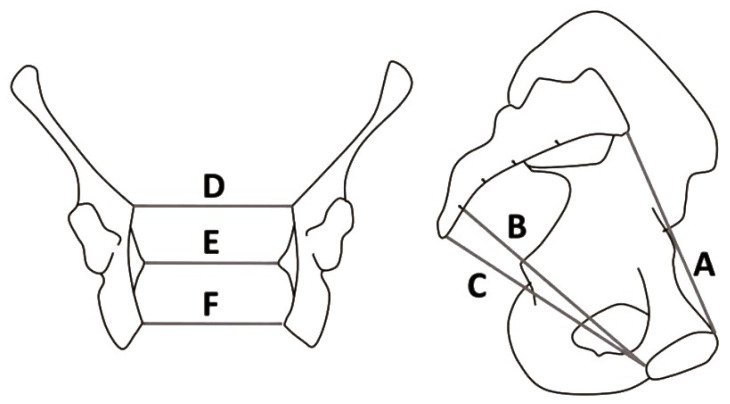
The human pelvis is organized and measured as follows. Three anteroposterior (A, B, C) and mediolateral planes create the birth canal (D, E, F): the inlet, the midplane, and the outlet, respectively (figure modified from Betti, 2017 [15]).

In the 1960s through the 1980s, the OD hypothesis was the most popular theory describing the difference between non-human primate and human birthing. Washburn generalized that non-human primates could birth “uncomplicatedly”, requiring little to no assistance to have a successful birth [1]. Conversely, humans often require multiple attendees to facilitate a safe and successful delivery. To understand the complexities of the human birthing process, the non-human primate and human pelvises must be understood. In non-human primates, the birth canal is shorter and less tube-like, described as a ring. This structure is less complex than the human canal, as it provides more space for the fetus to pass [13]. In humans, the measurement differences between various axes within and the canal’s shape are described as a truncated bent cylinder [14]. The human pelvis is structured with three planes that form the birth canal: the inlet, midplane, and outlet (Figure 1) [15]. The female pelvis has a wider sagittal dimension and transverse plane than the male pelvis [16]. In humans, the baby moves through the birth canal with a complex series of movements known as the “seven cardinal movements of labor” [17]. Therefore, measurements on the order of millimeters affect birth, considering the rotations a baby undergoes to get through the canal, which can pose more difficulties that could cause pathological issues for the mother, such as urogenital fistulas and levator ani tearing.

In primates in general and humans in particular, pelvis shape is sexually dimorphic and changes over time according to estradiol levels; the size of the pelvis changes to “prime” for birthing when a female reaches reproductive age [18]. Although the obstetric dilemma stated this difference as a locomotor and obstetric tradeoff compared to male pelvises, it is suggested that after a female reaches 40 years of age, the female pelvis becomes increasingly male-like [18]. Males do not have pelvic changes over time, and pelvis growth and development are somatic from sex-influenced autosomal genes causing hormonal changes [18]. These short-term changes cause expansion and reversal patterns to change the distance between ischial spines and are suggested to be the cause of obstructed labor. The bony structure is vital in supporting and protecting the abdominopelvic cavity, as it serves as an anchor point for tissues and muscles. Specifically, the levator ani muscle, which is critical for support of the pelvic organs, attaches to the ischial spines and is necessary to support the abdominopelvic cavity, especially during pregnancy [19].

In hominins—the taxonomic group including all humans, including our ancestors after they split from the chimpanzee lineage—evidence shows that bipedalism occurred before encephalization, and the cranial capacity of *Homo* has increased substantially in the last 600,000 years [20]. The pelvis size is disproportionate to the size of the human fetus, as the cranial capacity and body size are not much different from other newborn primates [21]. The human fetus, in relation to its mother’s weight, is twice as large compared to primates of similar size, making it an outlier for female human capacity. Additionally, human infants have accelerated brain growth outside the womb, known as secondary altriciality, where human brains are 30% less developed than other primates when birthed [22,23]. The demands of the size of the womb favored fetuses in earlier development in order to fit through the narrow birth canal; therefore, a fetus’s head grows significantly after birth [23].

## 3. Metabolic, Nutrition, and Socioeconomic Factors

Environmentally, nutrition contributes to obstructed labor, as micronutrients are crucial for good skeletal development and are linked to a successful pregnancy. Food access can depend on one’s culture, upbringing, socioeconomic status, and availability of healthy food. The term “food desert” is used to describe an area with little access to healthful food. It is important to note that communities with less access to healthy foods are at a higher risk of having compromised bone development [24]. Additionally, a study analyzing women who delivered at Loyola University Medical Center showed that the odds of having at least one morbid condition in pregnancy increased for patients living in food deserts [25]. Unfortunately, nutritional access has its own ethnic disparity, as African Americans are 38% more likely to report living in a food desert [26].

An essential nutrient supporting skeletal development is vitamin D. During the emergence of agricultural practices during the Neolithic Revolution, deformities in pelvises were prominent due to weak and poorly mineralized bone. Pre-agricultural diets were high in protein and low in carbohydrates, but farming introduced a major reduction in the diversity of consumed food, and post-agricultural methods turned people to a low-protein, high-carbohydrate diet [13]. Several bioarcheological studies note that locations such as the Middle East and Europe during the Neolithic Revolution experienced nutritional deficiencies and a resultant decrease in stature [27,28,29].

**Figure 2:**
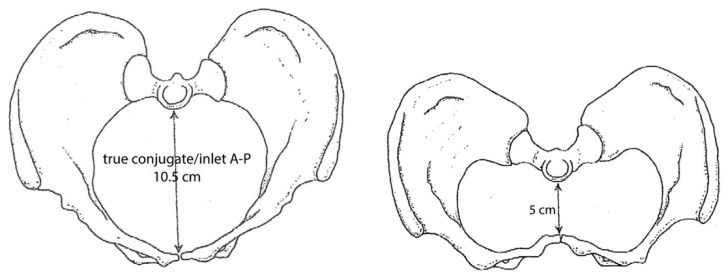
A normal-sized pelvis (**left**) compared to a contracted pelvis (**right**) (figure modified from Loudon, 1997 [34]).

Evidence shows that calcium and vitamin D deficiencies are associated with a higher risk of cesarean sections [30]. Severe vitamin D deficiency leads to rickets and osteomalacia in adults, and during the Industrial Revolution, people had low sunlight exposure and a diet lacking diversity. Consequently, osteomalacia and rickets were the most severe risk factors for maternal death until the 20th century; pelvises were deformed, and the shape did not provide a canal that the baby could easily pass through [31,32]. The most severe cases led to rachitic pelvises, characterized by deformities that made the birth canal misshapen, making it difficult or impossible for the fetus to exit the birth canal. Rickets allowed the body’s weight and the pull of the muscles to produce a pelvis that was flat anteroposteriorly [33,34]. Figure 2 shows a comparison of a normal pelvis shape and a contracted pelvis impacted by rickets [6].

**Figure 3:**
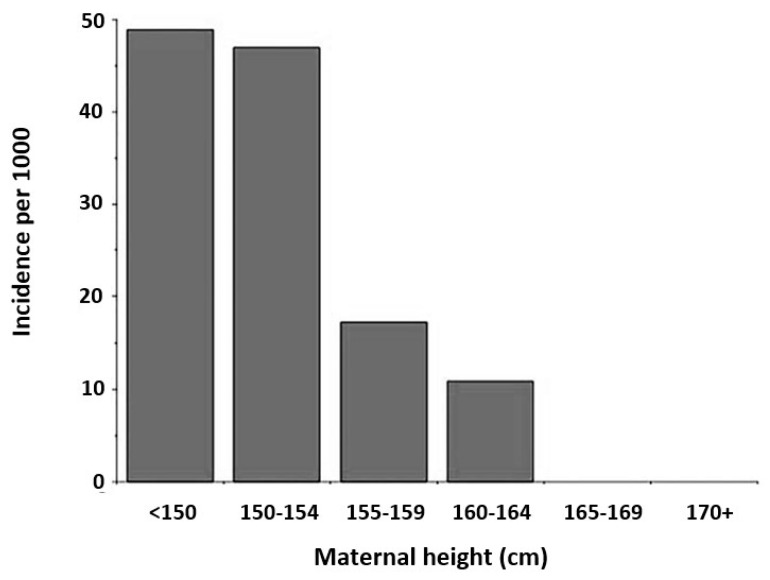
Association between incidence of intrapartum cesareans per 1000 deliveries and maternal height among women in Ouagadougou, Burkina Faso (figure modified from Wells, 2017, with data from Sokal et al. 1991 [36]).

Micronutrient deficiencies are still prominent in influencing both maternal and fetal growth. Mothers who grew up experiencing undernutrition showed shorter stature and improper pelvic growth, and short stature has a known association with smaller and flatter pelvises [35]. The association between a higher risk of obstructed labor and shorter maternal height was identified in a study completed in Burkina Faso (Figure 3) [36,37]. Mothers below the 20th percentile for height show an increased risk of cephalopelvic disproportion [37,38,39].

In regard to overnutrition, there is a complex association between obesity and income. In women studied in the United States, obesity prevalence decreased among women with increased educational levels and average income levels [40]. Those who identified as men had a more complex association. In contrast, women had a clearer distinction based on their education and income levels relative to the federal poverty level [40].

Obesity is prevalent in the United States, affecting 41.9% of its population (2017–2020), increasing from 30.5% in 2000 [41]. Disparities in obesity are noticeable between ethnic groups. In the United States, there was no association between a lower prevalence of obesity in African American women and increased educational and income levels, whereas non-Hispanic “white” women had a generally negative correlation trend between education and income [40]. In minority populations, an inverse relationship has been found between obesity and household income, with supporting research showing that minority ethnic groups are more likely to experience multidimensional poverty than their “white” counterparts [40,41]. Therefore, obesity is being revealed as a disease resulting from the intersection of ethnicity, socioeconomic status, accessibility to resources, and structural environment. 

Worldwide, some mothers experience the dual burden of being “stunted-obese”, where they were raised in a state of undernutrition, which led to growth stunting at first, but later experienced a rapid shift to overnutrition and obesity. Although it is assumed that better living conditions improve health outcomes and decrease labor difficulty, the overnutrition of these stunted mothers increases the fetus size and, therefore, fetopelvic disproportion [42]. General improvement in living conditions can increase access to nutrition for a growing fetus, which can grow larger than the mother’s birth canal, and on a population level, there could possibly be a general increase in obstructed labor due to access to calories [43]. Typically, obese mothers give birth to larger babies, and metabolic complications from obesity can contribute to delivery complications [23,44]. Notably, shorter and obese women are at an increased risk for gestational diabetes, which can lead to macrosomia in fetuses, leading to a tighter fit [45]. There is also evidence that improved living conditions worldwide have increased height, and for every 1 mm increase in height per year, the CS rate is predicted to grow by more than 10% because the fetus is bigger relative to the mother, despite the mother’s height increase [46]. The stunted-obese phenomenon has been shown in populations like immigrants in the United States and under-resourced communities, but there may be regions in the United States with American-born citizens who are disproportionately affected by this stunted-obese phenomenon, highlighting disparities in communities arising from childhood [43,47]. It would not be a surprise to find that the stunted-obese phenomenon disproportionately affects ethnic communities, as more African Americans live in food deserts, which have a correlation with increased obesity [48]. The intersection of ethnicity, poverty, and health outcomes profoundly impacts African American, Native American, and Latino communities. Socioeconomic disadvantages contribute to higher incidences of obesity, gestational diabetes, and other conditions that complicate childbirth and increase maternal morbidity [48]. This may further play a role in the wide maternal health gap between minority women and “white” women.

## 4. Genetics, Culture, Racism, and Pelvic Shape

Genetics are thought to impact fetopelvic disproportion due to heritability rates for factors that influence the size of the mother’s pelvis and the fetus. A twin study evaluating genetic and environmental influences on pelvic morphology demonstrated heritability rates ranging from 0.5 to 0.8 in 6 of 8 pelvic measurements [4]. A 2015 study found that women with large-headed neonates tended to have wider pelvises and larger heads themselves [49]. In response to preventing major fetopelvic disproportion, the pelvises of women with large-headed neonates accommodate to allow for easier birth passage, showing considerable covariation in pelvis size [49]. The correlation between head circumference and stature was shown to have a regression value of r = 0.45 in females and a stronger correlation of r = 0.49 in males [49]. Females with a large head had a shorter sacrum that projected outward from the birth canal and a more circular pelvic inlet, as opposed to an oval pelvic inlet, on average, for smaller heads [49]. Historically, a rounder pelvic inlet is associated with the “ideal” shape for neonate accommodation in the pelvis during birth [50]. Although this study shows evidence for lessening the difficulty of delivery of a large-headed neonate, selection pressure to make the pelvis more accommodating for birth in the general population has, on average, not been shown to be present. Infants with large head circumferences are delivered by unplanned cesarean delivery at about double the frequency compared to infants with a normal-range head circumference, irrespective of their birth weight [51]. This statistic, however, should be questioned because head circumference measurements are prone to error for numerous reasons, a few of them being infant scalp edema, equipment limitations, hair density, and the time after birth when the measurement is performed. The increased risk of instrumental delivery can lead to more fetal complications than with high-birth-weight infants, where birth weight is influenced by environmental factors such as the mother’s diet [52].

However, it is now clear that several epigenetic factors, such as nutrition and related socioeconomic aspects, play a huge role in this complex topic, as discussed below. Importantly, another item that is often neglected in such evolutionary discussions on birth obstruction concerns cultural and political factors, including cultural racist and sexist narratives that are more prevalent in certain societies. For instance, data in the U.S. show that the highest overall rates of unplanned cesarean delivery are in African American women, followed by Asian women [53]. Additionally, low-risk African American and Asian mothers had higher CS rates than “white” mothers, despite being good candidates for vaginal delivery [12,54]. Globally, there are significant differences in the CS rate within different countries, some of them having much higher rates than neighboring countries. The global average CS rate increased by 19% between 1990 and 2018, with the largest increase occurring between 2000 and 2010 [9]. In the period between 1990 and 2018, three countries showed a greater than 50% increase in CS rates: Turkey, Andorra, and Egypt; the lowest rate of CS was in Africa [9]. When divided into categories of country “development”, the least “developed” countries, such as those in Sub-Saharan Africa, had only an increase of 8.6% in CS in the same 1990–2018 timeframe, compared to the average of 19% worldwide, showing a great disparity globally [9]. These least “developed” countries also had the highest rate of maternal mortality from obstructed labor over a similar time period [55]. If the mother has access to cesarean delivery, the reasons for having a CS vary. Personal concerns include fear of vaginal childbirth or the mother’s self-doubt about the ability to have a safe vaginal birth [56]. However, it appears that the rising CS rates are physician- and system-based causes [57]. A study completed in Chile found cesarean deliveries being practiced more in patients with private healthcare who were required to have an obstetrician, as opposed to doctors or midwives in public and academic hospitals [58]. Cesarean sections come with several risks, particularly in low- and middle-income countries where mortality and morbidity from CSs are estimated to be disproportionately high, and also generally increase the likelihood that the born babies will develop mucosal auto-immune diseases, like celiac disease and ulcerative colitis, and even type 1 diabetes and asthma [59,60,61,62]. CSs without a medical indication must be minimized, as there are serious medical complications, like the risk of uterine rupture and abnormal placentation [63]. CS rates are predicted to increase, and sequelae like the morbidity and mortality of the fetuses and mothers in these deliveries will follow [9].

Some experts argue that the use of midwives and obstetricians since the mid-20th century is a prime example of gene–culture coevolution. In this case, the selective pressure for a wider birth canal and, eventually, easier childbirth would have been greater without modern obstetrics, as the rate of fetopelvic disproportion increased by up to 10–20% since the widespread use of CS [64]. Although this amount is negligible in clinical care, the short amount of time over which this change occurred renders it remarkable [64].

Concerning the topic of racism and sexism, historically, several medical practitioners have subscribed to the idea that there are different human “races” biologically. In 1886, Turner published an at-the-time distinguished paper comparing pelvic measurements from several human populations to investigate ethnic differences and concluded that pelvic shape does vary across “human groups” [65]. Turner noted that Europeans mostly had a transversely oval canal, whereas other populations assumed a rounder pelvis. Because a rounder pelvis more closely resembled that of apes and, at the time, the belief in the racial superiority of white Europeans over other populations was common, the rounder pelvis was seen as “less departure from the usual mammalian form (Europeans)” [65]. It was concluded that pelvic variation was an “evolutionary trend” because the rounder pelvis shape indicated an “arrest in evolution from the ape form, the true anthropoid, to the perfect human form which is characteristically flat” [65]. We now know that this notion is incredibly flawed. There is no specific pelvic shape that definitively designates any particular “race”, although some pelvic shapes are more common in some ethnic groups over others [15].

**Figure 4:**
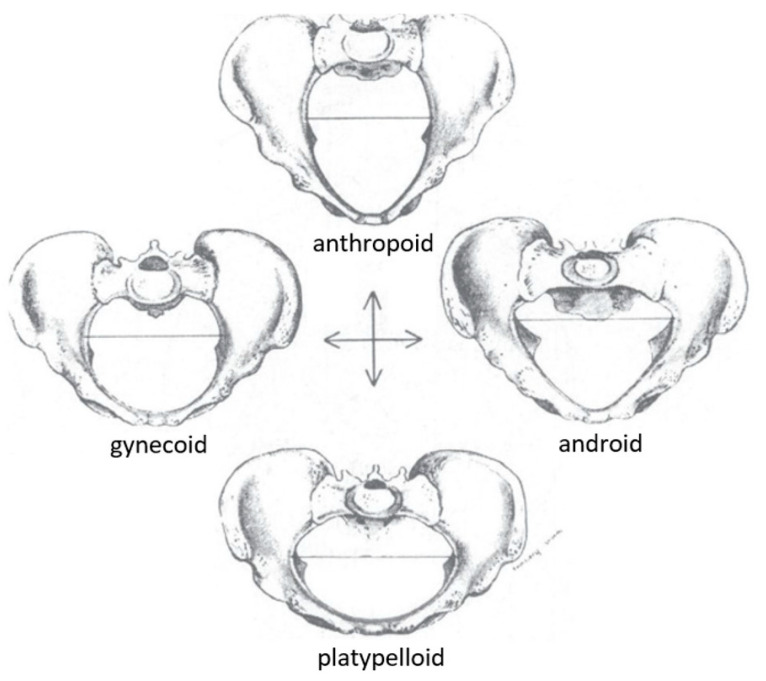
The four parent pelvis shapes coined by Caldwell and Moloy in 1938 (figure modified from Betti, 2021 [15].

Still, the early definitions of this race categorization can be unhelpful or harmful in the modern day. There are four categories of pelvis shape suggested by Caldwell and Moloy in the 1930s, and their model is the gold standard for obstetrical education today (Figure 4) [9,66]. The first is anthropoid, a long and narrow oval-shaped pelvic inlet. The second is platypelloid, characterized as a short and wide oval-shaped inlet. The third is gynecoid, a circle-shaped inlet flattened in the anteroposterior direction, and the fourth is an android-shaped pelvis with a heart-shaped inlet. In clinical practice, the gynecoid shape is favorable for birth due to its rounded shape. The gynecoid pelvis was considered the “normal female pelvis”, whereas the android pelvis resembled the male pelvis. However, it was recognized that there are mixed characteristics between these four classic types, known as borderline or mixed pelvis types of the four “pure” or “parent” forms [66]. The frequency of pelvic types in Caldwell and Moloy’s study is as follows. The gynecoid pelvis shape was most common, at around 42% of their sample. Anthropoid pelvis shapes were more common in African American women at 40.4%, compared to 23.5% of European women, and android pelvis shapes were prevalent in about 32.5% of European women and 15.7% of African American women [15].

The gynecoid pelvis is used to explain how humans evolved differently than apes and helps distinguish how and why the human birthing process is different from that of other primates. The explanation goes something like this—because the human pelvis shape required a more compact pelvic girdle so that humans could be bipedal, the fetopelvic ratio is smaller in humans compared to most apes. Humans, therefore, experience more difficulty in labor and have adopted a twisted birth canal so the fetus’s head will pass through with as much ease as possible. The canal starts with a transversely oval inlet and changes into an anteroposteriorly oriented outlet, therefore requiring the fetus’s head to rotate during labor and emerge facing backward or in the anterior-occiput position. In primate species, the pelvic opening is usually large enough for the fetus to pass through without requiring any rotations, and consequently, the fetus is delivered facing forward in the occiput posterior position [16]. Because the fetus is delivered facing forward in other primates, the mother can help extract the baby from the vagina by pulling it forward toward her, but humans cannot. Human mothers trying to extract their own babies certainly would risk damaging the child’s neck and spinal cord as the mother pulls the baby’s head backward. This explains why humans require obstetricians and midwives to assist with labor. The gynecoid pelvis and its associated mechanism of labor differ significantly from the anthropoid pelvis and its associated mechanism of labor. The anthropoid pelvis requires a different set of rotations and is more often associated with a forward-facing birth [67]. Because the gynecoid shape is used to describe how human labor came to be and is accepted as the model for women’s anatomy, it is unwittingly implying that the anthropoid pelvis and the mechanism of labor that accompanies it are “less typically human”, which is enormously racist [15]. The parallel between the words “anthropoid” and “animal” was not incidental; it instead reflected the intentions to debase African American individuals as anatomically deficient for the vital human act of giving birth [68].

Previously, the nineteenth and twentieth centuries characterized pelvis shape heavily around ethnicity. The European “race” was viewed as more evolved due to facing fewer difficulties in birthing compared to non-Europeans, characterized as “primitive people” by Engelmann in 1883 [69]. Physicians and anthropologists took the opportunity to create theories around the different pelvis shapes and how they impacted the ease of birth—often putting a harmful perspective on the evolution of people in general, as mentioned earlier [6]. Race and religious affiliation seemed to govern the difficulty of the birthing process and, ultimately, evolution itself [6]. For example, the French paleoanthropologist Rene Verneau allotted 18 pages to describing sexual dimorphism of the pelvis but 82 pages to racial comparisons of pelvic anatomy in his work at this time [70]. The gynecoid pelvis was the most observed in individuals with European genetic ancestry and was taught as the “norm”.

This topic relates directly to the practice of determining pelvis shape, which has been a method often used clinically to determine potential complications during birthing in the past 70 years. Clinical pelvimetry was used to evaluate pelvis morphology to assess the best delivery routes for complicated births, such as those in breech positions or arrested labor [14]. Pelvimetry could be performed radiographically or manually, with manual being invasive and, if implemented incorrectly, unhelpful to the mother’s and fetus’s outcomes [14]. In obstetrics, Yeomans nicely stated, “pelvic anatomy is to the obstetrician what abdominal anatomy is to the general surgeon—or should be” [14]. Yeomans does suggest the utility of classifying pelvis shape in deciding whether to continue with a CS or instrumental delivery, as the shape of the birth canal affects several aspects of labor, including fetal position and rotations in the canal, presentation at birth, and the likelihood of complications [15]. More recently, MRI pelvimetry measurements were used as predictive factors for emergent CS delivery in obstructed labor. Today, the sacral outlet diameter distance can be used in conjunction with clinical assessment when selecting a safe method of delivery, as it is seen as a more accurate depiction of fetopelvic proportionality, but other measurements, such as the bispinous diameter and inlet transverse diameter, are sometimes used [71].

Consequently, there is now a gap in education on the proper mechanisms and progression of labor in other pelvis types. “White” women are the basis of the “normal” gynecoid pelvis shape and obstetrics; therefore, education is largely shaped around this [72]. However, almost half of African American women have an anthropoid pelvis shape and have more surgical interventions, possibly contributing to morbidity and mortality [13,72]. Latino and Native American women are also among women with a higher prevalence of anthropoid and platypelloid pelvis shapes, which plays a role in their higher rates of cesarean sections [73]. Anthropoid pelvis shapes lead to generally uncomplicated labor; however, the rotations the fetus experiences differ from what is taught based on the gynecoid pelvis. There is proof that labor occurs differently between gynecoid and android pelvis shapes, but the prevalence in one group does not imply that one is normal and the other is not. A lack of acknowledgment leads to a lack of education, increasing the rate at which surgical procedures are used and increasing the rate of poorly performed interventions [13]. Forceps are often gently applied when birth needs to progress, and the gynecoid and anthropoid pelvis shapes require a different forceps approach. An attempt to rotate the fetus against the main canal diameter could lead to injury to the mother and baby or even stillbirth. The process of fetal rotation, especially when using forceps, can present specific risks due to the applied pressure and the possibility of misalignment. Manual rotation, while common in clinical practice, generally carries lower risks but still requires precision to prevent maternal or fetal injury. Forceps-assisted rotation, however, is associated with a higher potential for complications, including trauma to the fetal head or maternal soft tissue if not executed correctly [74]. Surgical interventions come with risks, including sepsis, hemorrhages, and thrombosis, the top causes of mortality and morbidity [75]. African American women have the highest rate of cesarean sections. One possible reason for this could be that African American women have a higher prevalence of hypertensive disorders and preterm gestation, which are strongly associated with elective primary Cesareans [76]. It is also not ruled out that poor provider–patient interactions or a mistaken belief about a patient by a provider may contribute to some African American women undergoing C-sections for reasons such as a misunderstanding of the patient’s pain threshold [76].

Another possible reason for this disparity could be the use of the vaginal birth after cesarean delivery calculator, which is the calculation used to make a prediction of how successful a woman with a previous CS will be in delivering vaginally. VBAC, or vaginal birth after C-section, is associated with decreased maternal morbidity, the avoidance of surgery and surgical complications, a lower risk of postpartum hemorrhage and infection, and a lower risk of complications in future pregnancies [77]. The likelihood of being selected as a candidate for VBAC, however, is lower for women who are African American or Hispanic, as one point is deducted from their overall calculated score for being one of these two ethnicities. The VBAC calculator was made to help providers individualize risk assessment for VBAC by accounting for personal risk factors but, unfortunately, has exacerbated ethnic disparities in maternal care and maternal outcomes. Only recently has a new VBAC calculator model that excludes race been proposed and used in practice, but unfortunately, it is not widely adopted [78].

The disparity that Native American pregnant women living on reservations face is unique but also echoes the theme that African American women face with poor physician–patient interactions and cultural misunderstandings. Early and continuous prenatal care is essential for providing proper education, discussing concerns, identifying high-risk patients, and implementing proper interventions to avoid negative birth outcomes. Unfortunately, Native American women seeking care through the Indian Health Service only begin their prenatal care in the first trimester 66.5% of the time, compared to other ethnicities that begin prenatal care in the first trimester 81.3% of the time [79]. Specifically, education on the cessation of substance use during pregnancy is helpful in reducing the rate of preterm delivery, infant mortality, and low birth weight, which are alarming statistics in Native American women. Native Americans have the highest rates of alcohol, marijuana, and drug use disorders compared to all other ethnic groups, necessitating the need for early pregnancy education and intervention in cases of drug use during pregnancy [80]. Living on the reservation offers a very low quality of life with high rates of poverty, sexual or physical abuse, and depression and substance abuse. A lot of Native American pregnant women in these environments receive little to no support from their partner or may be embarrassed by an unplanned pregnancy. In order to improve care, there is a need to understand why Native American women are significantly more non-adherent to prenatal healthcare. Some proposed ideas include cultural barriers, a lack of proper healthcare systems on reservations, and social determinants. Many Native American women express distrust with physicians, especially white physicians, and “Western medicine”. In addition, it is common for patients in the Indian Health Service to see a different physician at every prenatal appointment and wait 2 h for their 15 min prenatal appointment. Some even propose that a majority of care provided by natural helpers in the Native American community should take a stronger, official role in providing care in collaboration with clinicians [81]. Many minority groups experiencing similar struggles with healthcare may suggest that it is not just pelvic shape that plays a strong role, but rather the interaction of various factors contributes to maternal morbidity and mortality. Despite Native Americans having the widest pelvises, this group faces extremely poor maternal health outcomes [82]. This further suggests that a more encompassing Extended Evolutionary Synthesis view is needed to explain maternal morbidity and mortality.

Acknowledging these differences is normalizing the variation in pelvis shapes and understanding how to address it in practice. Even in areas where “white” women are a minority, books teaching the anatomy and obstetrics of primarily European women are donated from universities to communities in largely African American areas. This ultimately will influence how healthcare providers in that area care for the non-‘white’ population they serve [13]. Geographical proximity governs genetic and phenotypic resemblance in pelvis shape, and it is suggested that pelvis shape and size variations are less common than skin color variance geographically [13]. As modern society includes globalization and immigration, the teaching of one shape of the pelvis puts future mothers at a disadvantage in their pregnancy journey.

New improvements in technology to measure and assess pelvic shape as a factor in a safe delivery bring about new classifications. Humans immigrated from Africa around 60,000 years ago, which is not a significant amount of time to evolutionize into vastly different pelvic shapes. As stated earlier, the anthropoid pelvis shape is far more similar to other human pelvises than that of any ape [15]. More recent studies do not support the existence of four distinct pelvic types as defined by Caldwell and Moloy. In fact, the literature is now saying that pelvic anatomic variation within populations is more due to nutrition and climate than ethnicity [15]. A study in Australia used CT scans to measure 64 female pelvises and found no specific groupings but instead a spectrum of many different shapes [83]. It is very possible the Caldwell and Moloy classification is incorrect, as it was only made using the data of a few thousand pelvises and only in one specific region, the United States. The recent evidence that pelvic shape depends more on nutrition and climate than other factors may have skewed the data Caldwell and Moloy were pulling from to make their classification. Although limited scientific literature is published about this topic, there is a notion that assessing the pelvic type during the prenatal period is not useful in predicting cephalo-pelvic disproportion, as the external pelvic shape may not correspond to internal features of the pelvis, like the pubic arch, sacral prominence, and ischial spinous distance, which all play a stronger role in various mechanical issues associated with obstructed labor [84,85]. This has thankfully encouraged some obstetricians and midwives in the U.S. to abandon the Caldwell and Moloy classification system and adopt anti-racist methods of pelvic anatomy analysis. Students are now being taught that assessments are individualized and not tied to socially constructed race categories, as there is quite a broad and normal range of variation in pelvic shapes [84].

## 5. Conclusions

The evolution of the hominin pelvis has incited much debate about encephalization, bipedal locomotion, and the evolutionary progress of humans. Despite the high risk to the mother, labor has remained relatively the same, with variation in fetopelvic disproportion occurring due to a complex interaction between genetic, environmental, and cultural factors influencing the development of the female pelvis. The rate of encephalization compared to the capacity of the female pelvis brings a high risk of complications in vaginal delivery for both the fetus and mother. Evolution created a complicated birthing process—but environmental factors like nutrition and ethnic disparities have exacerbated the already-difficult process of vaginal birth. Historical data have shown that nutritional deficiencies have devastating impacts on maternal skeletal development, and current data show how overnutrition impacts fetal weight. Socially, understanding the historical, cultural, socioeconomic, and so-called “racial” basis for pelvis classification must be acknowledged, and this type of education should be revised. Clinicians should seek to redefine the “normal” pelvis shape to teach appropriate interventions to prevent complications in a range of patients. Acceptance of the variation in the female pelvis and its causes is imperative to reducing maternal morbidity and mortality for mothers and their babies. We have the opportunity to improve maternal outcomes for underserved communities, and a promising first start is to emphasize racism as a key determinant of illness and death. Ethnicity-conscious medicine, an agenda aimed to promote attentive, anti-racist practices over unchecked assumptions that uphold racial hierarchies evident in modern healthcare today, will push medicine into making the much-needed jump toward achieving health equity. The 2023 edition of *Varney’s Midwifery* has updated its pelvic classifications to highlight specific pelvic characteristics and the interactions between the fetus and the pelvis and leave the Caldwell and Moloy classifications in the past. This is promising, but only a small change is needed to uproot the U.S. obstetrical care system, which privileges a model of childbirth and anatomy chiefly based on women of European descent.

## Data Availability

Not applicable.
